# A randomized pilot study to evaluate graft versus fistula vascular access strategy in older patients with advanced kidney disease: results of a feasibility study

**DOI:** 10.1186/s40814-020-00619-9

**Published:** 2020-06-17

**Authors:** Mariana Murea, Randolph L. Geary, Denise K. Houston, Matthew S. Edwards, Todd W. Robinson, Ross P. Davis, Justin B. Hurie, Timothy K. Williams, Gabriela Velazquez-Ramirez, Benjamin Bagwell, Audrey B. Tuttle, Shahriar Moossavi, Michael V. Rocco, Barry I. Freedman, Jeff D. Williamson, Haiying Chen, Jasmin Divers

**Affiliations:** 1 Department of Internal Medicine, Section on Nephrology, Wake Forest School of Medicine, Medical Center Boulevard, Winston-Salem, NC 27157-1053 USA; 2 Section on Gerontology and Geriatric Medicine, Wake Forest School of Medicine, Winston-Salem, NC USA; 3 Department of Vascular and Endovascular Surgery, Wake Forest School of Medicine, Winston-Salem, NC USA; 4 Department of Biostatistics and Data Science, Division of Public Health Sciences, Wake Forest School of Medicine, Winston-Salem, NC USA; 5 Division of Health Services Research, Department of Foundations of Medicine, NYU Long Island School of Medicine, Long Island, NY USA

**Keywords:** Arteriovenous vascular access, Hemodialysis, Kidney disease, Older adults, Randomized trial

## Abstract

**Background:**

Although older adults encompass almost half of patients with advanced chronic kidney disease, it remains unclear which long-term hemodialysis vascular access type, arteriovenous fistula or arteriovenous graft, is optimal with respect to effectiveness and patient satisfaction. Clinical outcomes based on the initial AV access type have not been evaluated in randomized controlled trials. This pilot study tested the feasibility of randomizing older adults with advanced kidney disease to initial arteriovenous fistula versus graft vascular access surgery.

**Methods:**

Patients 65 years or older with pre-dialysis chronic kidney disease or incident end-stage kidney disease and no prior arteriovenous vascular access intervention were randomized in a 1:1 ratio to undergo surgical placement of a fistula or a graft after providing informed consent. Trial feasibility was evaluated as (i) recruitment of ≥ 70% of eligible participants, (ii) ≥ 50 to 70% of participants undergo placement of index arteriovenous access within 90 to 180 days of enrollment, respectively, (iii) ≥ 80% adherence to study-related assessments, and (iv) ≥ 70% of participants who underwent index arteriovenous access placement will have a follow-up duration of ≥ 12 months after index surgery date.

**Results:**

Between September 2018 and October 2019, 81% (44/54) of eligible participants consented and were enrolled in the study; 11 had pre-dialysis chronic kidney disease, and 33 had incident or prevalent end-stage kidney disease. After randomization, 100% (21/21) assigned to arteriovenous fistula surgery and 78% (18/23) assigned to arteriovenous graft surgery underwent index arteriovenous access placement within a median (1st, 3rd quartile) of 5.0 (1.0, 14.0) days and 13.0 (5.0, 44.3) days, respectively, after referral to vascular surgery. The completion rates for study-specific assessments ranged between 40.0 and 88.6%. At median follow-up of 215.0 days, 5 participants expired, 7 completed 12 months of follow-up, and 29 are actively being followed. Assessments of grip strength, functional independence, and vascular access satisfaction were completed by > 85% of patients who reached pre-specified post-operative assessment time point.

**Conclusions:**

Results from this study reveal it is feasible to enroll and randomize older adults with advanced kidney disease to one of two different arteriovenous vascular access placement surgeries. The study can progress with minor protocol adjustments to a multisite clinical trial.

**Trial registration:**

Clinical Trials ID, NCT03545113.

## Key messages

- What uncertainties existed regarding the feasibility?

No trial has objectively evaluated clinical outcomes in patients with advanced kidney disease based on the type of *first* hemodialysis arteriovenous vascular access placed. Hence, the feasibility of randomly assigning these patients to different surgical interventions for dialysis vascular access is unknown. Because this study focused on older patients with chronic disease, uncertainties existed regarding rates of eligibility, recruitment, retention, and adherence to the surgical intervention.

- What are the key feasibility findings?

Results of this pilot study showed acceptable rates of participant eligibility, consent, adherence to the intervention, and retention. The pilot reports 81.5% recruitment rate, 88.6% surgical intervention rate with median time to index arteriovenous access surgery of 7.0 days, and 6.8% drop-out rate and 11.4% mortality rate at a median follow-up of 215.0 days. The protocol required amendments to several inclusion criteria. Respondent burden to study-specific assessments was noted, with variable completion rates of assessments of physical function and quality of life.

- What are the implications of the feasibility findings for the design of the main study?

Results will guide the research team to select numbers of participating clinical sites for the main study. Certain inclusion criteria in the pilot study (i.e., age cut-off and pre-dialysis chronic kidney disease) and exclusion criteria (i.e., prevalent end-stage kidney disease) have been refined or removed to optimize assessment of outcomes. Initial age-based inclusion criterion was adults 70 years and older; the criterion was changed to adults 65 years and older. Initial ESKD-based criterion excluded patients with prevalent ESKD; this exclusion criterion was removed to include patients with prevalent ESKD who otherwise met all eligibility criteria. Variability in time to post-operative assessment between the intervention groups was noted and recognized that it could introduce confounding on intervention effects. An unexpected improvement in grip strength over time was also noted. We surmise that a selective dropout process may be at play, which could bias the observed results. For the main trial, the frequency of assessments will be changed to reduce participant fatigability and rates of missing data. Time-points of post-operative outcome assessments will be set at definite intervals from the date of surgical intervention. In the main trial, statistical analyses to assess longitudinal changes in grip strength and other quantitative outcomes will treat death as a competing event.

## Background

### Introduction

Surgical creation of an arteriovenous (AV) fistula is considered the optimal long-term hemodialysis vascular access strategy in patients with pre-dialysis chronic kidney disease or dialysis-dependent end-stage kidney disease. Surgical placement of an AV graft is reserved for those who do not have vasculature suitable for placement of an AV fistula. For almost two decades, the standard practice of fistula-first arteriovenous access placement stemmed from observational studies in younger patients and was bolstered by subsequent practice guidelines [1–5].

Prior retrospective studies showed a graded relationship between the type of vascular access placed (or utilized) for hemodialysis with access complications and patient survival. The lowest access complication rates and longest patient survivals were seen with an AV fistula. The highest access complication rates and shortest patient survivals were seen with tunneled central venous catheters; intermediate results were seen with an AV graft [1–4]. The benefits of an AV fistula over an AV graft and catheter in these studies led national dialysis and vascular access committees to develop clinical practice guidelines. These guidelines, in place from 1996 through 2019, advocated an orderly approach to vascular access placement designated as the “Fistula First Catheter Last” initiative [5]. Nationwide efforts to increase placement and use of AV fistula for hemodialysis with this formulaic approach led to rapid declines in AV graft placement. Therefore, many patients are forced to use a catheter at dialysis initiation, as the success of AV fistula development is dependent on patient-specific factors [6, 7].

Flaws exist in the AV fistula-centered practice strategy. “Fistula First” practice guidelines were based on research performed in the 1990s, an era when older patients comprised less than 10% of patients with end-stage kidney disease. Remarkable changes in demographics (rising mean ages) and morbidities (rising prevalence of diabetes, cardiovascular disease, and malignancy) have since occurred in incident and prevalent dialysis populations. Based on national registry data encompassing the dialysis population in the early period of 2010s, adults ≥ 65 years comprise more than 40% of incident patients with end-stage kidney disease on hemodialysis [8]. Compared with their younger counterparts, older patients on dialysis have greater numbers of concurrent illnesses, polypharmacy, undertreated conditions, hearing impairment, and a high prevalence of physical and mental disabilities [9–11]. The prognosis for older adults initiating hemodialysis is poor, 25-60% mortality rates are seen in the first year (compared with 5-15% in their younger counterparts) [12–14]. Recent studies showed that frailty and morbidity index directly impact patient survival on dialysis, independent of the type of vascular access [9, 15–17].

A limitation in the long-used “Fistula First” vascular access guidelines related to translating conclusions from observational data into clinical practice. A challenge in all retrospective studies is distinguishing whether the intervention (here, vascular access type) directly impacted clinical outcomes or merely accentuated the effects of other comorbidities leading to observed outcomes. Patients who undergo AV fistula placement and achieve a functional access may be healthier, yet this may not be reflected in their list of medical problems (e.g., perceived better prognosis, less severe comorbid conditions) [18, 19]. Residual confounders not only impact achievement of a usable AV fistula but also affect patient survival. In a large cohort from the US Renal Data System comprised of incident patients ≥ 67 years old, those who initiated HD with a catheter and had a history of primary AV fistula failure (i.e., underwent AV fistula placement surgery and the fistula did not mature) experienced 34% lower mortality rates than those who initiated hemodialysis with a catheter but did not undergo AV fistula placement surgery, despite both groups of older incident dialysis patients receiving hemodialysis via a central venous catheter [18]. These results support the contention that patient factors affecting the outcome of fistula placement may also impact mortality on hemodialysis. The revised vascular access guidelines recognized these limitations and no longer state that all patients should have an AV fistula as a preferred vascular access [20]. The 2019 guidelines moved the focus away from the “Fistula First” approach and urged providers to think not only about what AV access is first but also about what is next during the planning of the first access, emphasizing having a P-L-A-N for each patient: Patient Life-Plan first, followed by his or her corresponding Access Needs [21]. Given these fundamental gaps in knowledge, prospective unbiased data are urgently needed to compare patient outcomes between AV fistula and AV graft in older patients initiating hemodialysis.

Apart from age-related differences in clinical outcomes, age-related biological factors can have an independent effect on vascular access outcome. Several studies highlighted the challenging task of achieving a functioning AV fistula in older patients [22]. In a cohort of 168 patients who underwent AV fistula placement, the 12-month primary assisted patency was 35% in older patients (≥ 70 years) group and 67% in younger patients (< 70 years) group (*P* = 0.002); secondary patency was 36% and 67%, respectively (*P* = 0.004) [23]. A meta-analysis of 13 studies concluded that older patients have 50-65% higher odds of primary AV fistula failure and 80% higher odds of secondary AV fistula failure compared with younger patients [24]. These results led to the emerging concept that an AV graft may represent a better “catheter-sparing” strategy than AV fistula, because grafts often permit faster transition from catheter-based to AV access-based dialysis. This must be balanced against reports from prior retrospective analyses of shorter AV access lifespan of an AV graft (median cumulative patency 7.0 months) vs. an AV fistula (median cumulative patency 15.0 months) [19], although one study reported similar AV fistula and AV graft access survival in patients 75 years of age and older [25].

Given the paucity of clinical trials data in the field of AV vascular access placement, we conducted a randomized pilot study to investigate the feasibility of implementing randomization to AV fistula vs. AV graft placement strategy in older patients with advanced kidney disease. This manuscript reports data that directly addressed the feasibility and acceptability of trial procedures. Effectiveness is not reported, because the pilot study was not powered to detect outcome differences between study arms. Feasibility and pilot studies are designed to evaluate trial feasibility, acceptability, and safety, rather than test the effectiveness of planned main trial interventions [26, 27].

## Study objectives

1. The primary objective of this pilot trial was to examine the feasibility of random assignment to AV fistula or AV graft vascular access placement in older patients with advanced chronic kidney disease with no prior AV access intervention. Participants were referred for surgical intervention for AV access creation by their nephrologist and had vascular anatomy suitable for placement of either an AV fistula or AV graft. Study procedures and estimates of the rates of study eligibility, consent, randomization, and retention were evaluated.
2. The secondary objective of this pilot trial was to analyze the feasibility of data collection related to assessment tools of physical function and activities of daily living, by observing completion rates, and missing data.

## Methods

The design and rationale of the trial have been published previously [28]. The eligibility criteria and schedule of assessments were adjusted during the pilot phase of the trial. A brief description is presented below.

### Setting and participants

Following research ethics approval by an Institutional Review Board (IRB), this pilot study was conducted at 16 outpatient dialysis units, one inpatient dialysis unit, and three nephrology practices affiliated with an academic tertiary hospital and a regional hospital in North Carolina, USA. The nephrology practices in this region serve an annual population of nearly 500 prevalent patients with end-stage kidney disease on hemodialysis. All adult patients with pre-dialysis chronic kidney disease or incident end-stage kidney disease who were referred for AV access placement by their treating nephrologist were assessed for eligibility.

### Study design

Eligible patients were randomized in a 1:1 ratio either to AV graft placement (intervention group) or to AV fistula placement (control group). The randomization sequence was computer-generated, and allocation was managed by the Research Coordinator of the Department of Internal Medicine, Section of Nephrology, Wake Forest School of Medicine

### Inclusion

- Age ≥ 65 years
- Incident or prevalent end-stage kidney disease on long-term hemodialysis
- Pre-dialysis chronic kidney disease expected to require hemodialysis initiation within 90 days of screening
- No previous surgery for AV access placement
- Tunneled central venous catheter as the sole vascular access used for hemodialysis
- Medically and surgically eligible to undergo placement of either AV fistula or AV graft access

### Exclusion

- Native vasculature not suitable for placement of AV fistula or AV graft access based on clinical evaluation or ultrasound-guided vein mapping
- Imminent kidney transplant (expected within 6 months of screening)
- Inability to provide informed consent
- Life expectancy < 1 year

Both upper extremities were studied with duplex ultrasound to assess brachial and radial artery size, blood vessel quality (e.g., calcification), and blood flow characteristics. Cephalic, basilic, and brachial veins were assessed for caliber and for pathology (e.g., thrombus and wall thickening). Central venous flow was assessed for spontaneous and phasic flow to rule out central venous stenosis. Patients were required to have a cephalic vein without disease and a diameter of 2.5 mm or greater at the wrist or antecubital fossa, or a basilic vein of greater than 2.5 mm from elbow to upper arm to be considered eligible for an AV fistula. For an AV graft, brachial artery and vein diameters of 3 mm or greater were required without significant disease. The nondominant arm was preferred after randomization when vascular anatomy was suitable in that extremity for the assigned procedure (AV fistula or AV graft). Extremities were excluded if vascular imaging suggested inflow artery stenosis or significant artery disease at the site of proposed anastomoses. Extremities were also excluded if a pacemaker defibrillator with central venous leads was present on that side.

### Recruitment

Potential participants were first screened at the time of initiation of hemodialysis or at referral for AV access placement, whichever occurred first. The decision for referral to vascular access surgery was made by treating nephrologists, independent from this study. Review of electronic clinical records at outpatient dialysis facilities, nephrology outpatient clinics, and nephrology inpatient service determined eligibility for the study. Eligibility for anesthesia and surgical placement of an AV access was determined as part of standard of care in each patient. Surgical suitability for placement of AV access was determined by the vascular surgeon using the results of the ultrasound vascular mapping of both upper extremities. Final eligibility criteria encompassed medical and surgical eligibility for placement of either type of AV access. Once all inclusion and exclusion criteria were met, eligible participants were approached and the study rationale and objectives were explained in lay language. Written informed consent was obtained in person.

### Randomization and concealment

Consenting participants underwent baseline study-specific assessments followed by randomization to either AV graft or AV fistula access placement. Block randomization, with variable block size of 2, 4, and 8, was used with no stratification. Following randomization, each participant’s assignment was conveyed to the vascular surgery team on the day of randomization and on the day prior to surgery. Blinding of the research team or the participant to the type of AV access placed was not possible. However, vascular access outcomes (i.e., primary AV access failure, access-related infections) were adjudicated by patients’ medical and/or surgical team, independent of the study team, and without consideration of participant assignment.

### Trial intervention

Based on randomization, participants were scheduled to undergo surgical placement of AV graft or AV fistula. Surgical creation of the AV access was performed by a vascular surgeon with experience in dialysis vascular access placement. All AV graft were made of polytetrafluoroethylene. When suitable vasculature was present, preference was given to distal (forearm) over proximal (arm) AV access placement in both study groups to preserve limited future vascular access sites.

### Data collection

Patient demographics, medical history, vascular anatomy and surgery details, and postoperative complications were extracted from the electronic medical record. Data were de-identified and collected by a trained study coordinator. When applicable, clinic notes, operative reports, procedure notes, and discharge summaries were reviewed to ensure capture of all events. Study-specific assessments to evaluate participant’s upper extremity muscle strength, physical activity, level of independence, satisfaction with AV access, and health-related quality of life were performed as described [28].

### Trial feasibility—participant recruitment and retention rates

Patients referred by their Nephrologist for AV access placement were approached for study participation at their outpatient assessment by the vascular surgeon. Eligible participants were approached for recruitment by either the study coordinator and/or the vascular access coordinator and/or the study PI and/or the vascular surgeon. Screen failure logs and enrollment logs were maintained for all patients screened and approached for participation. Reasons for eligibility failure were recorded and entered into an Excel spreadsheet. Recruitment success was defined as ≥ 70% of eligible participants agreeing to be enrolled. Successful retention was considered as ≥ 70% of participants who underwent AV access placement will have a follow-up duration of ≥ 12 months after index access placement.

### Trial feasibility—barriers to the implementation of the intervention

Reasons for not undergoing AV access surgical intervention were recorded. Intervention success was defined as ≥ 50 to 70% of participants undergoing placement of index AV access within 90 to 180 days of enrollment, respectively.

### Trial feasibility—collection of outcome data

Inpatient (hospitalization records) and outpatient (dialysis units, office visits) electronic medical records were reviewed on a monthly basis to collect events of vascular access outcome (e.g., primary or secondary AV failure, date of first cannulation, date of successful cannulation, angioplasty, AV access infection, catheter-related infection, etc.) and patient outcomes (e.g., hospitalization, surgical intervention). All medical and/or surgical diagnoses and vascular access-related diagnoses documented in this study were made by the participant’s treating physicians. Outcome measures based on interviewer-administered questionnaires and grip strength and gait speed measurements were obtained at four-time points with in-person visits done by the study coordinator pre-operatively on the day of surgery (baseline assessment) and pre-dialysis at the outpatient dialysis unit (follow-up assessments). The baseline assessment occurred at study enrollment, with further assessments performed 2 weeks after index AV access placement (follow-up 1), 6 weeks after the first cannulation of the index AV access (follow-up 2), and 6 months after successful use of index AV access or 6 months after index AV access placement if primary AV access failure occurred (follow-up 3). Feasibility of data collection was defined as ≥ 80% participant adherence to study-specific assessments (grip strength, level of independence, satisfaction with vascular access, and health-related quality of life), absent of a condition that precluded assessment.

### Trial feasibility—adverse events

Adverse events collected in this pilot study encompassed access-related events, hospitalizations, and death. Access-related adverse events included nerve injury, AV access infection, bleeding from AV access site requiring intervention, and arterial steal syndrome with or without required intervention. Adverse events were collected prospectively through a monthly review of inpatient and outpatient electronic medical records. Clinical diagnoses and causes of death were excerpted from the chart and recorded based on the conclusions of the treating physicians. The event was recorded in detail and reported to the IRB, when appropriate. An independent medical safety officer, unblinded to treatment assignments, reviewed participant clinical outcomes and vascular access-related events on a quarterly basis; sooner, if indicated.

### Clinical outcomes assessment

Grip strength was measured using a hand-held dynamometer on a non-dialysis day or pre-dialysis during a dialysis day. A cut-off point of <16 kg in women and < 26 kg in men was used to define muscle weakness [29]. Functional independence was assessed at baseline and follow-up 3 using the instruments of activities of daily living (ADLs) and instrumental ADLs (IADLs) [30, 31]. Health related quality of life was assessed at baseline and follow-up 3 using the kidney disease quality of life short form questionnaire (KDQOL-SF) version 1.3 [32]. Pain at the AV access site from access cannulation was assessed at follow-up 1, follow-up 2, and follow-up 3 using the verbal descriptor scale (VDS) [33]. The study protocol was amended to include measurement of gait speed at baseline, follow-up 2 and follow-up 3 using 4-meter gait speed (4MGS) test (amendment 3). Patient satisfaction with the vascular access was assessed follow-up 1, follow-up 2, and follow-up 3 using the short-form vascular access questionnaire (SF-VAQ) [34].

### Statistical analysis

This feasibility study was designed to test interventions and estimate the proportions of participants who would meet future objectives in a well-powered multisite randomized clinical trial. Demographic and participant characteristics are presented using mean (standard deviation) or median (1st, 3rd quartile) for continuous variables and number (percent) for categorical variables. Descriptive statistics were used to report feasibility outcomes. Recruitment and retention rates, adherence to the trial intervention, barriers to the implementation of the intervention, and the feasibility of collecting outcome assessment data are summarized and reported as frequencies and proportions or as free text. Results are considered based on the CONSORT extension to pilot trials [35]. Between groups, inferential comparisons were not performed as the study was not powered for this analysis.

### Ethical considerations

Confidentiality of patient data was maintained throughout the study and case report forms were kept in locked cabinets. The electronic data extracted from the monitoring systems was anonymized, as was all data in the final reports.

## Results

### Screening

**Fig. 1:**
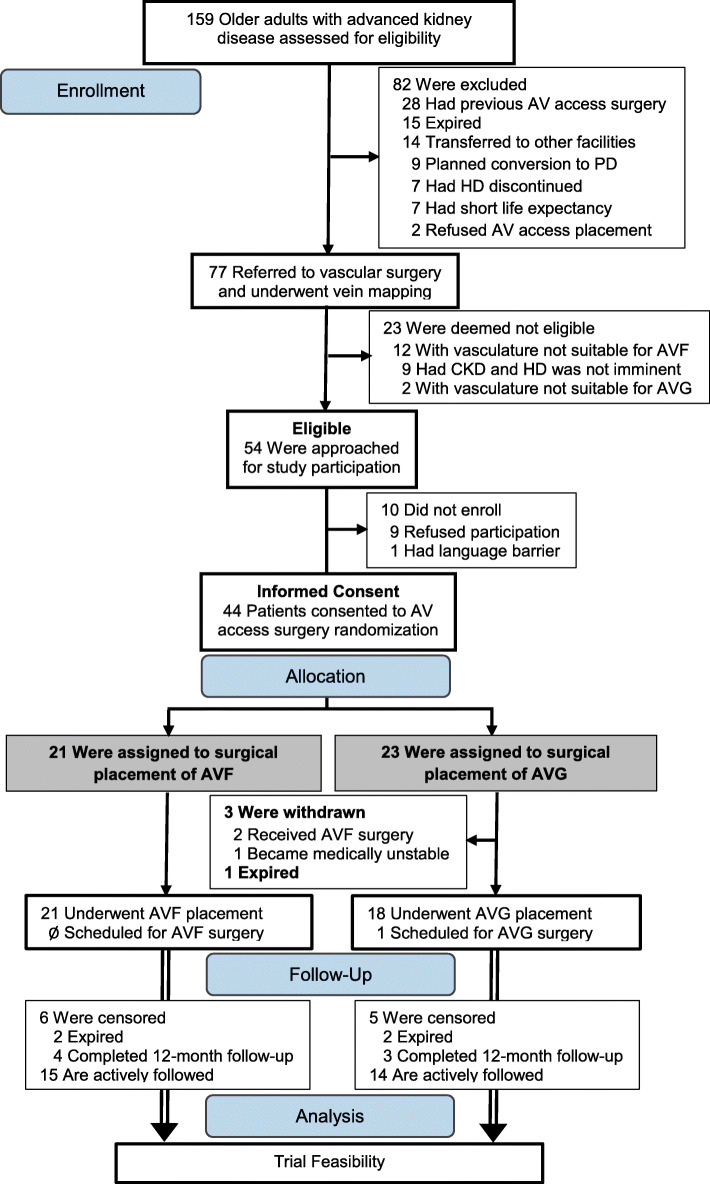
Study flow diagram. Screening and randomization between Sept 01, 2018 and October 30, 2019

Figure 1 shows participant flow through the trial. Between September 1, 2018 and October 30, 2019, 159 older adults received care for advanced chronic kidney disease at a study site. Of these, 82 were ineligible due to previous AV access placement surgery (*n* = 28), planned conversion from hemodialysis to peritoneal dialysis (*n* = 9), transfer of nephrology care outside study sites (*n* = 14), death before referral for AV access placement (*n* = 15), discontinuation of renal replacement therapy (*n* = 7), short life expectancy (*n* = 7), or refusal of surgical placement of an AV access (*n* = 2).

### Eligibility rate and reasons for exclusion

The eligibility rate was calculated by dividing the number of patients referred to vascular surgery for AV access placement by the number who met the inclusion criteria after ultrasound vascular mapping of both upper extremities. This equated to an eligibility rate of 70.1%. Out of the 77 older adults with advanced chronic kidney disease who were referred to vascular surgery and underwent vein mapping, 14 (18.2%) did not have vasculature suitable for placement of AV access, and 9 (11.7%) were felt unlikely to require initiation of hemodialysis within 90 days.

### Consent rate and reasons for not participating in the study

**Fig. 2:**
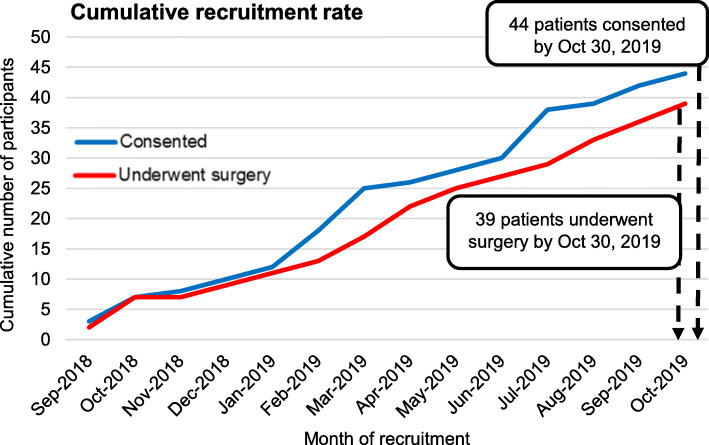
Cumulative recruitment rate

The overall consent rate was 81.5%. Of the 54 patients eligible for this study, 44 agreed and consented to participation. Nine (16.7%) declined to participate with reasons for refusal being apprehension to undergo placement of an AV access other than an AV fistula (*n* = 7) or general wariness of study participation (*n* = 2). The average enrollment rate was 3 patients per month (Fig. 2). Baseline characteristics of the participants are listed in Table 1. The median age of the recruited patients was 76.3 years (1st, 3rd quartile, 70.8, 81.8), 17 (38.6%) were female and 30 (68.2%) were white.

**Table 1:** Baseline characteristics of the study participants

Characteristic	All	AVF-first	AVG-first
Total participants (*n*)	44	21	23
Age (years)	76.3 (70.8, 81.8)	78.9 (70.7, 81.1)	72.8 (71.1, 82.0)
Sex, female, *n* (%)	17 (38.6)	6 (28.6)	11 (47.8)
Race or ethnic group, *n* (%)			
Black	14 (31.8)	7 (33.3)	7 (30.4)
White	30 (68.2)	14 (66.7)	16 (69.6)
Body-mass index	27.9 (24.7, 33.7)	25.5 (23.6, 28.4)	30.9 (26.7, 35.6)
Kidney disease status, *n* (%)			
CKD	11 (25.0)	7 (33.3)	4 (17.4)
ESKD	33 (75.0)	14 (66.7)	19 (82.6)
Coexisting medical conditions, *n* (%)			
Diabetes and complications of diabetes	31 (70.5)	16 (76.2)	15 (65.2)
Hypertension	39 (88.6)	19 (90.5)	20 (87.0)
Myocardial infarction	7 (15.9)	2 (9.5)	3 (13.0)
Heart failure	10 (22.7)	6 (28.6)	4 (17.4)
Peripheral arterial disease	9 (20.5)	6 (28.6)	3 (17.4)
Stroke	12 (27.3)	5 (23.8)	7 (30.4)
History of tumor without metastases	11 (25.0)	5 (23.8)	6 (26.1)
Chronic pulmonary disease	6 (13.6)	4 (19.0)	2 (8.7)
Moderate or severe liver disease	2 (4.5)	1 (4.8)	1 (4.3)
Dementia	4 (9.1)	3 (14.3)	1 (4.3)

Baseline data were collected at the time of patient enrollment. Data are presented as number of participants (percentage) or median (1st, 3rd quartile). Race or ethnic group was self-reported. The body-mass index is the weight in kilograms divided by the square of the height in meters.* AVF* arteriovenous fistula; *AVG* arteriovenous graft; *CKD* chronic kidney disease; *ESKD* end-stage kidney disease

### Randomization procedures

Twenty-one participants were randomized to AV fistula placement and 23 to AV graft placement. The distribution of demographic characteristics and coexisting comorbidities are shown in Table 1

### Intervention adherence

Of the 44 participants randomized and scheduled to receive index AV access placement, 34 underwent surgery as scheduled and 10 missed their first appointed surgical date (4 in fistula group and 6 in graft group). Of those who missed initial appointments, 9 had their surgical date rescheduled and one patient in the graft group had access surgery canceled due to medical issues. Of the 9 patients with rescheduled surgery, 7 underwent surgery on their rescheduled date and 2 missed their rescheduled surgical date and were scheduled for a third time to receive index AV access placement. The adherence rate to surgical intervention was 100.0% in the AV fistula group and 78.2% in the AV graft group. At the time of data lock for reporting of results, 21 of 21 participants in the fistula group underwent placement of AV fistula, 18 of 23 participants in the graft group underwent placement of AV graft, and index AV access surgery was pending in one participant in the graft group. The overall adherence rate was 88.6% with 95% confidence (79.3%, 98.0%) with both bounds greater than 70%. This suggests the intervention appears to be feasible in terms of patient adherence rates. Surgical interventions for index AV access placement are summarized in Table 2. Median time to index AV access surgery calculated from date of referral to date of surgery was 7.0 days (2.0, 26.0). The 9 participants who had surgery rescheduled underwent index AV access placement at a median of 90 days (26, 112) after initial surgical appointment date. Of the total 39 vascular access surgical interventions for placement of index AV access, 31 (79.5%) were to create the AV access on the left upper extremity and 28 (71.8%) located the index AV access in the forearm.

**Table 2:** Vascular access interventions

Characteristic	All (*n* = 39)	AVF-first (*n* = 21)	AVG-first (*n* = 18)
Time to index AV access surgery*, days	7.0 (2.0, 26.0)	5.0 (1.0, 14.0)	13.0 (5.0, 44.3)
AV access location			
Left forearm	32 (82.1)	12 (57.1)	10 (55.6)
Left arm	10 (25.6)	6 (28.6)	4 (22.2)
Right forearm	3 (7.7)	1 (4.8)	2 (11.1)
Right arm	4 (10.3)	2 (9.5)	2 (11.1)
Patients with rescheduled AV access surgery	9 (23.1)	4 (19.0)	5 (27.8)

Data are reported as median (1st, 3rd quartile) or number of patients (percentage)

*AV* arteriovenous; *AVF* arteriovenous fistula; *AVG* arteriovenous graft; *HD* hemodialysis

^*^Time to placement of index AV access was calculated from the date of vascular surgery referral to date of surgical intervention

### Retention rate

Three (6.8%) patients, all in the graft group, were withdrawn from the study (1 became medically unstable to undergo surgery and 2 had protocol deviation and received AV fistula placement) (Fig. 1). At the time of data lock, 7 participants had completed 12 months of follow-up, 5 participants had expired, and 29 were actively followed. Among the 5 participants who expired, 2 were in the fistula group and expired after index AV surgery; and 3 were in the graft group, of whom 1 expired before and 2 expired after index AV access surgery (Fig. 1). The observed median time to death was 168.0 days (127.0, 210.2) from the date of enrollment. The feasibility benchmark for retention rate required that ≥ 70% of participants who underwent index AV access placement will have a follow-up duration of ≥ 12 months after index access placement. Based on these preliminary results, we observed an overall drop-out rate of 6.8% and a mortality rate of 11.4% within a median follow-up of 215.0 days (111.3, 282.0). Overall, these results suggest that we are on target of meeting the feasibility aim for participant retention rate.

### Completion rate

**Fig. 3:**
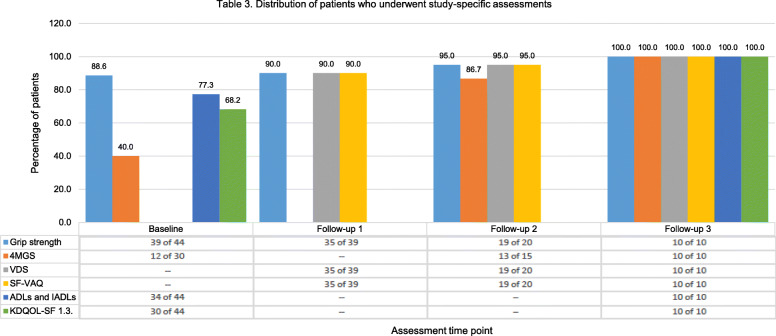
Distribution of patients who underwent study-specific assessments

Figure 3 shows the completion rate for all study-specific assessments expressed as the participants who completed the assessments out of assessment-eligible participants. Note the denominator (assessment-eligible participants) used to calculate the completion rate for each questionnaire, grip strength and gait speed measurement varied based on the requirements to meet the assessment (i.e., time from first cannulation of the index AV access, time from successful use of the index AV access, number participants enrolled following the amendment for gait speed measurement). Instruments assessing ADLs/IADLs and KDQOL-SF 1.3. were completed by 77.3% and 68.2% of participants at baseline and all who reached the follow-up 3 assessment time point. The completion rate for 4MGS at baseline, follow-up 2 and follow-up 3 was 40.0%, 86.7%, and 100.0%, respectively. The completion rate for VDS/SF-VAQ at follow-up 1, follow-up 2, and follow-up 3 was 90.0%, 95.0%, and 100.0%, respectively. The estimated time (minutes) required to complete each study-specific assessment was 4 min for grip strength, 7 min for 4MGS, 4 min for VDS, 4 min for SF-VAQ, 8 min for ADLs, 12 min for IADLs, and 20 min for KDQOL-SF 1.3.

### Outcome assessment

At the time of data lock for this report, participants had a median follow-up duration of 188.0 days (86.0, 269.5). Primary AV access failure was documented in 13 (33.3%) of all 39 index AV accesses placed. By type of first AV access placed, primary access failure occurred in 8 (38%) of 21 AV fistulas and 5 (27.8%) of 18 AV grafts. Successful cannulation of the index AV access occurred in 11 (28.2%) participants, 6 (28.6%) in the fistula group and 5 (27.8%) in the graft group. Median time to successful AV access cannulation calculated from date of access placement was 122.5 days (83.0, 172.5) in patients with AV fistula and 54.0 days (51.0, 56.0) in patients with AV graft.

**Fig. 4:**
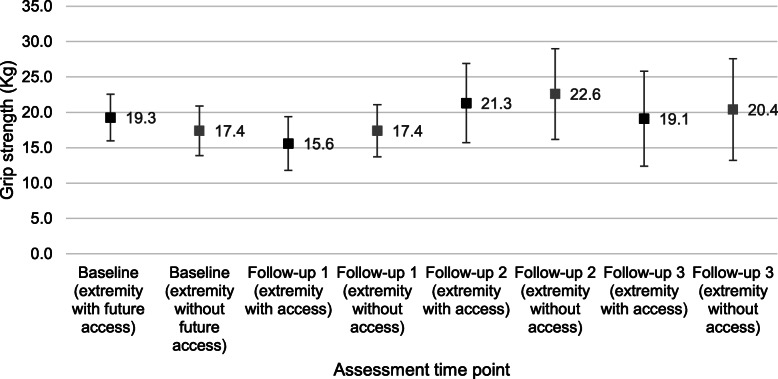
Distribution of grip strength, mean (95% confidence intervals), at baseline (in the upper extremity that will receive the index AV access [black mark] and the contralateral upper extremity [gray mark]) and during follow-up (in the upper extremity that received the index AV access [black mark] and the contralateral upper extremity [gray mark])

Grip strength was measured in > 85% of participants who reached pre-determined assessment time points. At baseline, 5 of 44 enrolled patients did not have grip strength tested due to the presence of peripheral intravenous lines in the upper extremities. At the first follow-up, 14 days after the surgery, 4 of 39 patients who underwent AV access placement did not have grip strength measured due to pain in the upper extremity related to the surgical wound. One of 20 patients did not have grip strength measured at follow-up 2 due to joint pain. Figure 4 shows grip strength distributions in both upper extremities at baseline and in the upper extremity without and with AV access measured on follow-up. Baseline mean (95% confidence level) grip strength in the upper extremities was 19.3 kg (3.3) and 17.4 kg (3.5). Post-operatively, grip strength in the upper extremity with and without AV access was 15.6 kg (3.8) and 17.4 kg (3.7) at follow-up 1, 21.3 kg (5.6) and 22.6 kg (6.4) at follow-up 2, and 19.1 kg (6.7) and 20.4 kg (7.2) at follow-up 3, respectively.

Eighteen of 30 participants at baseline and 2 of 15 at follow-up 2 did not have gait speed assessed due to being wheelchair bound or exhibiting unsafe walking. At baseline, 10 and 14 of 44 participants declined assessment of ADLs/IADLs and KDQOL-SF 1.3., respectively, reportedly due to the burden of evaluation.

### Adverse events

Five deaths were observed, 2 in the fistula group and 3 in the graft group (Table 3). One patient randomized to AV graft placement expired before surgical intervention (patient D in Table 3). One patient with chronic kidney disease who underwent AV fistula placement was not started on hemodialysis by the date of expiration (patient B). Of the 4 participants who underwent index AV access placement and expired, none had AV access-related infection and one had a catheter-related infection.

**Table 3:** Summarized description of the 5 participants who expired during the study period

Patient	Kidney disease status at enrollment	Randomization (AVF or AVG)	Underwent index AV access placement (Yes or No)	AV access successfully cannulated (Yes, No, or N/A)	Event of AV access-related infection (Yes, No, or N/A)	Event of TCVC-related infection (Yes, No, or N/A)
A	ESRD	AVG	Yes	Yes	No	No
B	CKD	AVF	Yes	N/A	No	N/A
C	ESRD	AVG	Yes	No	No	Yes
D	ESRD	AVG	No	N/A	N/A	No
E	ESRD	AVF	Yes	Yes	No	No

Kidney disease status reported at the time of AV access placement. Days to expiration date reported related to date of AV access surgery, date of HD initiation, and date of successful AV access cannulation (when applicable)

*AV* arteriovenous, *HD* hemodialysis, *TCVC* tunneled central venous catheter, *N/A* not applicable

### Lessons learned

This pilot trial was very informative. The investigative team learned a number of things that will help in conducting a full trial. The lessons gleaned from this pilot study, along with a summary of 14 methodological items that will be evaluated in a feasibility study are listed in Table 4 [36].

**Table 4:** Summary of findings against 14 methodological issues for feasibility research

Methodological items	Findings	Evidence
1. What factors influenced eligibility and what proportion of those approached were eligible?	We identified modifiable and unmodifiable factors for inclusion eligibility.	Modifiable factor: inclusion age and incident ESKD. Changing the age cut-off and enrolling patients with prevalent ESKD augmented the rate of recruitment. Unmodifiable factors: previous AV access surgery, poor prognosis, expired before surgical referral, and vasculature not suitable for fistula or graft placement. Other: nephrology care located outside research sites (14 out of 159 patients screened). Involvement of other clinical sites will enhance recruitment.
2. Was recruitment successful?	Yes. Recruiting success was defined as ≥ 70% of eligible participants agreeing to participate and enroll in the study. Revised of inclusion and exclusion criteria facilitated recruitment.	A total of 81.5% of eligible participants agreed to participate and were enrolled in the study.
3. Did eligible participants consent?	Yes.	Forty-four (81.5%) out of 54 eligible patients consented to participate.
4. Were the participants successfully randomized?	Yes. Randomization procedures worked well.	Differences between the AVG-first group (intervention) and AVF-first group (control) are expected as may occur with small sample size.
5. Were blinding procedures adequate?	No. Blinding to intervention did not occur.	Blinding the study team members (e.g., PI, study coordinator, vascular surgeon) to the type of AV access placement was deemed infeasible due to the visible nature of the intervention.
6. Did participants adhere to the intervention?	Yes. Successful adherence to the intervention was defined as at least 80% of the participants receiving the AV access according to randomization arm.	Thirty-nine (88.6%) out of 44 patients randomized to AV access placement underwent surgery and received the index AV access (78.3% in the AVG group and 100% in the AVF group).
7. Was the intervention acceptable to the participants?	Yes. Acceptability was assessed as refusal of eligible patients to consent.	Nine (16.7%) out of 54 eligible patients refused study participation.
8. Was it possible to calculate intervention costs and duration?	An economic evaluation was not conducted as part of this study.	N/A
9. Were outcome assessments completed?	Reasons for non-completion of the questionnaires or 4MGS test included evaluation burden or inability to ambulate safely.	The completion rate for baseline and follow-up assessments of grip strength was 88.6-100.0%. Between 22.7-31.8% of the participants reported questionnaire fatigue and declined completion of baseline assessments for ADLs, IADLs, and/or KDQOL-SF 1.3. The protocol was amended to include 4MGS; for this test, we noted that about 60.0% of the participants could not have gait speed assessed due to severe baseline functional impairment.
10. Were outcomes measured those that were the most appropriate outcomes?	All outcomes were deemed appropriate and valid.	The main clinical outcomes included AV access primary failure rate, time to successful cannulation, and access-related complications.
11. Was retention to the study good?	Attrition rate was assessed as the proportion of participants who were consented for the study and then withdrawn or censored at transfer to other facility or refusal to remain in the study.	Four (9.1%) of the 44 participants were withdrawn from the study.
12. Were the logistics of running a multicenter trial assessed?	Yes. This pilot trial spanned across 16 dialysis units, two hospital centers (one academic tertiary center and one regional hospital), and three nephrology practices.	Overall, the logistics and study procedures were adequate and functional in most areas, and important insights were gained to inform the design and efficient conduct of the planned future multisite trial. These include the following: allowing a realistic timeframe for regulatory approval and site start-up; clear communication between the PI and stakeholders of different sites (nephrologists, vascular surgeons, vascular access coordinators, study coordinators, and dialysis unit managers); establish clarity over inclusion/exclusions and methodological approach; obtain approval and establish organization for performing study-specific assessments at the outpatient dialysis units; employment of a range of strategies to retain trial center engagement.
13. Did all components of the protocol work together?	The main components of both the study protocol and intervention worked well together.	Trial methodology—two-stage screening, evaluation of vein mapping results to determine final eligibility, and correspondence with the surgical team—was streamlined during this pilot trial.
14. Did the feasibility/pilot study allow a sample size calculation for the main trial?	No. A sample size calculation for a future RCT was not calculated	Our feasibility study did not provide a meaningful effect size estimate for planning a subsequent RCT. This is due to the imprecision inherent in data from small sample sizes.

## Discussion

This pilot trial assessed feasibility in preparation for a multisite randomized clinical trial to compare effectiveness and safety between the two hemodialysis AV access placement strategies. The primary outcome in the main trial will be the rate of catheter-free days following index AV access placement and the safety outcome will be the rate of access-related infections.

Surgical referral for placement of an AV vascular access and type of vascular access in patients with advanced chronic kidney disease remain fundamental clinical and scientific conundrums due to lack of high-quality data guiding these practices. This is particularly true in older populations, where available data suggests different outcomes compared to younger patients. However, current practice does not consider age-related personalized care in hemodialysis vascular access type.

Health care interventions require rigorous evaluation and this is best achieved with randomized controlled trials. Feasibility studies are an important initial step in the phased approach to identifying problems that might occur in planned main clinical trials for complex interventions in complex populations. In this case, the trial would consider the placement of dialysis AV vascular access in older adults with advanced chronic kidney disease and multiple comorbidities. We therefore performed an important preparatory stage analysis by conducting a feasibility study to ensure methodological approaches applied in a future multisite clinical trial are streamlined. Herein, we report the feasibility results of a pilot study for a first of its kind randomized clinical trial investigating placement of AV fistula versus AV graft in adults 65 years of age and older with pre-dialysis chronic kidney disease or end-stage kidney disease receiving hemodialysis via a catheter.

This pilot study achieved a 100% screening rate. At initiation, one large academic group and 14 dialysis centers encompassed the recruitment sites. Six months into the study, participating sites were expanded to include 2 additional nephrology practices, 2 additional dialysis units, and one regional hospital. Logistical adaptations were promptly implemented such that study personnel were able to screen all older adults who initiated chronic hemodialysis and/or were referred for vascular access placement at all participating sites. We observed two events of protocol deviation wherein participants underwent placement of AV fistula instead of randomization-based placement of AV graft. To prevent the occurrence of similar protocol deviations in the full-scale trial, AV access assignment will be reminded to the vascular surgery team and conveyed to the operating room nursing personnel the day on the day of surgical intervention.

Eligibility rates inform whether those recruited are likely to be representative of the target population, with higher eligibility rates suggesting generalizability of the study. In this pilot trial, the eligibility rate was 70.1%. A trial parameter that indicates potential loss in precision, which can introduce bias and reduce statistical power, thereby affecting the generalizability, validity, and reliability of results is loss to follow-up. It has been estimated that a ≥ 20% loss can threaten trial validity [37]. The retention rate in this pilot study was good, with 6.8% drop-out rate. However, completion rates of questionnaires and gait speed were suboptimal. Throughout the study, our research team discussed their observations about how participants acted during the assessment. Easy fatigability and refusal to perform certain tests were noted. These issues are likely due to the chronically ill and frail nature of participants. To remedy this issue, the order of assessments was reorganized by reducing the frequency and number of questionnaires.

Besides changing the frequency of post-operative assessments for the main trial, the time-points of assessment will be set at definite periods from the date of surgical intervention. In the design of the pilot trial, the time-points of second and third assessments (follow-up 2 and follow-up 3) depended on other outcomes (i.e., first cannulation and successful use of index AV access). As a result of this pilot trial, we appreciate that differences in time to AV access cannulation between the intervention groups could introduce systematic confounding of outcome assessment. Therefore, assessment visits will be set at fixed intervals after the date of surgical intervention during the main trial to eliminate potential confounding between the timing of measurement and treatment effect. This pilot trial also notes a rather unexpected improvement in grip strength over time, with higher average grip strength recorded at follow-ups 2 and 3 compared to baseline grip strength. Survival bias due to healthier participants (i.e., fewer comorbidities, better baseline physical status and grip strength) being more likely to reach later assessment time points could skew the interpretation of grip strength outcomes. In the main trial, analyses will be run under the intention-to-treat principle, and the effect of missing data and informative censoring will be assessed using appropriate imputation techniques and sensitivity analyses. Death as a competing risk will also be assessed in addition to the proposed death-censored outcomes.

While the recruitment rate was acceptable considering the frail and ill population involved, we recognize it will take more than 4 years to reach the required sample size given rates of recruitment from the two participating sites. Originally, we planned to recruit patients ≥ 70 years of age with incident end-stage kidney disease. The eligibility criteria were adjusted to include adults ≥ 65 years of age, as well as those with pre-dialysis chronic kidney disease referred for AV access placement. We subsequently recognized that many vascular access-related outcomes (e.g., primary AV access failure, time to successful AV access cannulation) would not be assessed in a timely manner for patients who were not on hemodialysis at the time of AV access placement. Therefore, we plan to limit enrollment in the main trial to those with end-stage kidney disease receiving maintenance hemodialysis via catheter at the time of referral for vascular access placement.

Additional factors that may affect AV access development will be taken into consideration in the full-scale clinical trial. The literature has suggested that the site of AV access placement (forearm vs. arm) and sex may influence access patency. An analysis of 941 AV fistula accesses (median follow-up, 23 months) stratified by configuration showed that AV fistula placed in the forearm (i.e., radiocephalic accesses) had lower 12-month primary patency and maturation rates compared with AV fistula placed in the arm (i.e., brachiocephalic accesses) (30% vs. 44%, *P* = 0.005) in patients aged 65 years and older; differences in AV fistula outcomes by anatomical location were not seen in the non-old adults < 65 years of age [38]. In a retrospective study based on a national cohort 9458 patients ≥ 67 years of age, started on hemodialysis with a catheter who underwent AV fistula or AV graft placement within 6 months of dialysis initiation, females had higher adjusted likelihoods of unsuccessful AV fistula use (hazard ratio [HR] 1.46, 95% CI 1.36-1.56), assisted AV fistula use (HR 1.34, 95% CI 1.17-1.54), and AV fistula abandonment (HR 1.28, 95% CI 1.10-1.50) than males [39]. In the main clinical trial, randomization will be stratified by sex to ensure balanced representation in both arms. Exploratory analyses will include testing for interaction effects between AV access configuration (forearm vs. arm) and treatment assignment (AV fistula vs. AV graft) to determine whether the treatment effect on the primary outcome is modified by AV access anatomical location.

## Limitations

Our trial has many strengths and several limitations. The pilot was conducted in only two hospital centers (a large academic center and a regional hospital) across three nephrology practices and 16 dialysis units. Additional logistical limitations are expected to be encountered with a large multisite randomized clinical trial. Going forward, potential research sites will be evaluated to ensure resources will be available to timely address any logistical issues that may arise. Another limitation was the slow rate of enrollment, complicated by reductions in the number of potential participants due to high mortality in this chronically ill population. This observation will help us select the number of sites necessary to participate in a large-scale well-powered trial.

Implementation of the randomization process in the pilot study was not free from potential bias. Allocation to the two arms of surgical interventional was not concealed from the study coordinator at the time of approaching potential participants. However, patients were not informed of their randomization arm until informed consent was obtained. To reduce potential bias in the implementation of the randomization process in a future main trial, randomization will be implemented using a web-based system that performs randomization once the informed consent was obtained. The random sequence will be coded, implemented, and executed by a web-based system to provide a random assignment that cannot be changed. Blinding to the type of index AV access placed during the study was not feasible due to the visible nature of the intervention. This limitation will remain in the main clinical trial. However, clinical outcomes (e.g., primary AV access failure, access-related infections, and hospitalizations) are adjudicated by treating physicians and are unlikely to be influenced by knowledge of study participation. For the main trial, the study coordinators recruiting patients and the statistician at the analysis stage will be blinded to the type of AV access intervention. In addition, the main clinical trial will encompass oversight committees (i.e., data safety and monitoring board, endpoint adjudication, and steering committees) to address efficacy and/or safety endpoints in an unbiased manner.

## Conclusions

The results from this pilot study provide the feasibility of conducting a randomized clinical trial to test the impact of two type of AV access placement on clinical outcomes, physical function, and quality of life in older patients with end-stage kidney disease on hemodialysis. The pilot trial provided an indication of the rates of recruitment, refusal, eligibility, randomization, and retention which should be expected in a full-scale trial. Overall, the high screening rate, eligibility rate, retention rate, and adherence to intervention indicate that the study approach has internal and external validity. Rigorous assessment of the effects of AV fistula versus AV graft strategy in older adults with end-stage kidney disease on health outcomes and goal-concordant care will require an adequately powered prospective clinical trials.

## Data Availability

The datasets used and/or analyzed during the pilot study will be provided upon request to the principal investigator (Dr. Mariana Murea) by approved researchers.

## Abbreviations

ADLs: Activities of daily living
AV: Arteriovenous
4MGS: Four-meter gait speed
KDQOL-SL 1.3.: Kidney disease quality of life short form questionnaire version 1.3.
IADLs: Instrumental ADLs
SF-VAQ: Short-form vascular access questionnaire
VDS: Verbal descriptor scale
